# Generating a Single Session Outcome Measure from Digital Mental Health Platform Footprints Using Natural Language Processing

**DOI:** 10.23889/ijpds.v8i3.2269

**Published:** 2023-09-11

**Authors:** Gregor Milligan, Aynsley Bernard, Liz Dowthwaite, Elvira Perez Vallejos, James Goulding

**Affiliations:** 1 N/LAB, University of Nottingham; 2 Horizon CDT, University of Nottingham; 3 Kooth PLC, London; 4 Horizon Digital Economy Research, School of Computer Science, University of Nottingham; 5 School of Medicine, University of Nottingham

## Introduction & Background

This work demonstrates the development of an Adult Session Wants and Needs Outcome Measure (Adult SWAN-OM) aimed at supporting service delivery within the digital mental health platform (DMHP), Qwell. Qwell is a DMHP commissioned by the United Kingdom’s National Health Service which provides access to an online community of peers, a team of experienced counsellors, and a cadre of emotional well-being practitioners. The service is anonymous at point of entry and free for users, and provides an extensive, person-centred approach which results in a wide range of user needs. Deriving outcome measures from the platform’s varied counselling sessions, aims to provide both insights into the want and needs of users and underpin improved mental health support.

## Objectives & Approach

The objective of this research is to show how contemporary machine learning methods (Transformer Models and Contextualised Topic Modelling) may be combined with digital footprint data (in the form of seldom explored text data generated on DMHPs) to identify service user wants and needs. Specifically, with automated inference of patient outcomes currently scarce, we focus on the development of outcome measures in the context of ‘single sessions’, applying machine learning methods to extract topics related to the wants and needs of service users on DMHPs.

## Relevance to Digital Footprints

The data used in this study consisted of transcripts between Qwell practitioners and service users (SU’s) (N=874) at conversation level (N=2323), a filter was applied to the dataset to ensure that focus the SUs elicitation of wants and needs fit into the criteria of a single session. Individuals in the final selected cohort (n=192) are not significantly different from the wider Qwell SU population in the study period in terms of age, gender or ethnicity; suggesting that the cohort is representative of the wider target population. This study shows the potential of mental health digital footprints data when providing insight into the wants and needs of DMHP SUs.

## Conclusions & Implications

Via this analysis of mental health digital footprint data, this work establishes a process for creating a new outcome measure through the computational analysis of transcript data, incorporating insights from clinical experts and individuals with lived experience of engaging with DMHPs with textual data analysis. This methodological approach of Transformer Models and Contextualized Topic Modelling enables the analysis of a considerable volume of data faster than manually reviewing transcripts. We offer suggestions for the refinement of automated methods, in collaboration with direct support and feedback from both clinicians and individuals with lived experience of DMHPs to enable the understanding of wants and needs of service users within DMHPs.

